# The effect of precision and power grips on activations in human auditory cortex

**DOI:** 10.3389/fnins.2015.00378

**Published:** 2015-10-15

**Authors:** Patrik A. Wikman, Lari Vainio, Teemu Rinne

**Affiliations:** ^1^Institute of Behavioural Sciences, University of HelsinkiHelsinki, Finland; ^2^Advanced Magnetic Imaging Centre, Aalto University School of ScienceEspoo, Finland

**Keywords:** audiomotor integration, attention, auditory cortex, precision grip, power grip

## Abstract

The neuroanatomical pathways interconnecting auditory and motor cortices play a key role in current models of human auditory cortex (AC). Evidently, auditory-motor interaction is important in speech and music production, but the significance of these cortical pathways in other auditory processing is not well known. We investigated the general effects of motor responding on AC activations to sounds during auditory and visual tasks (motor regions were not imaged). During all task blocks, subjects detected targets in the designated modality, reported the relative number of targets at the end of the block, and ignored the stimuli presented in the opposite modality. In each block, they were also instructed to respond to targets either using a precision grip, power grip, or to give no overt target responses. We found that motor responding strongly modulated AC activations. First, during both visual and auditory tasks, activations in widespread regions of AC decreased when subjects made precision and power grip responses to targets. Second, activations in AC were modulated by grip type during the auditory but not during the visual task. Further, the motor effects were distinct from the present strong attention-related modulations in AC. These results are consistent with the idea that operations in AC are shaped by its connections with motor cortical regions.

## Introduction

According to a prominent hypothesis, posterior parts of the superior temporal plane (STP) support general action-to-perception functions during audiomotor tasks (Warren et al., 2005; Hickok and Poeppel, 2007; Zatorre et al., 2007; Rauschecker and Scott, 2009; Rauschecker, 2010). Human functional magnetic resonance imaging (fMRI) studies have shown that these areas, particularly the planum temporale (PT), are activated during tasks requiring overt sound localization, vocalization, and playing of a musical instrument (Buchsbaum et al., 2001; Wise et al., 2001; Hickok et al., 2003; Chen et al., 2006, 2008a,b; Baumann et al., 2007). These findings support the role of posterior STP in guiding motor behavior based on auditory information.

Previous studies in animals and humans have also shown that responses in auditory cortex (AC) to the subject's own voice are suppressed during vocalization (Curio et al., 2000; Houde et al., 2002; Eliades and Wang, 2003; Christoffels et al., 2007; Flinker et al., 2010; Greenlee et al., 2011; Agnew et al., 2013). Such suppression is thought to be initiated by modulatory signals from motor cortices providing predictive information about the expected auditory input (Christoffels et al., 2007; Reznik et al., 2014). However, a recent study showed that excitatory neurons in the rat AC are suppressed before, and during a wide range of natural movements that are not related to vocalization, such as locomotion and head movements (Schneider et al., 2014). This suggests that motor execution may also modulate operations in AC when the motor task is not directly associated with vocal sound production.

Activations in AC are also strongly modulated by attention (Hall et al., 2000; Petkov et al., 2004; Rinne et al., 2005; Woods et al., 2009; Rinne, 2010; Alho et al., 2014). Attention-related modulations are typically equal or even greater in magnitude than activations elicited by the presentation of sounds. Further, AC activations depend on the characteristics of the attention-engaging task. For example, discrimination and memory tasks performed on identical sounds are associated with different distributions of activations along the superior temporal gyrus (STG; Rinne et al., 2009, 2012; Harinen and Rinne, 2013). As motor manipulations can easily be associated with changes in attention or task demands, these factors have to be carefully controlled in studies on audiomotor processing.

The present fMRI study was designed to investigate whether manual responses modulate activations in human AC (motor regions were not imaged). Our subjects were presented with identical stimuli during auditory and visual task blocks. The stimuli consisted of pairs of pitch varying tones and Gabor gratings with varying orientation. During auditory task blocks, subjects were required to ignore the visual stimuli and detect target sound pairs with increasing or decreasing pitch among non-target pairs with no pitch change. At the end of each block, they reported whether there were more targets with a rising or falling pitch. Depending on the task instruction, subjects also responded immediately after detecting a target or gave no overt motor responses during the block. In the visual task blocks, subjects performed analogous tasks on Gabor gratings with the target being a clockwise or counterclockwise orientation change. During the visual task, they were to ignore the auditory stimuli, report the relative number of targets at the end of each block, and respond to targets according to task instruction.

The visual conditions allowed us to investigate AC activations to sounds in the absence of directed auditory attention and, in particular, whether AC activations to sounds are modulated by motor responses (to visual targets) that are not associated with the auditory stimuli. The auditory conditions, in turn, were used to investigate the general effect of auditory task on AC activations (vs. visual task with identical stimuli and motor responses) and to examine whether manual motor responses (to auditory targets) modulate AC activations to the attended sounds.

The control of precision (i.e., the thumb and fingertips are used for manipulation of small objects, e.g., a pencil) or power grips (i.e., the whole hand is used to manipulate bigger objects, e.g., a hammer) may involve distinct neural networks and interact with the processing of sensory information (Ehrsson et al., 2000; Grézes et al., 2003). For example, precision and power grip responses are facilitated by a viewed object or by a heard syllable if the size of the object or the pitch of the syllable is congruent with the grip (i.e., small object/high pitch for precision grip; large object/low pitch for power grip; Tucker and Ellis, 2001; Makris et al., 2013; Vainio et al., 2014). Correspondingly, preparing a precision or a power grip response facilitates the perception of an object if its size is congruent with the prepared grip (Symes et al., 2008). Therefore, in the present study, we also investigated whether the possible effects of manual responses on AC activations depend on the grip type.

Our primary hypothesis was that motor responding would interact with auditory processes, which would be manifested particularly as suppression of AC activations to sounds. In addition, it was anticipated that AC activations could be differently modulated depending on whether subjects use precision or power grips to respond to targets.

## Materials and methods

### Subjects

Subjects (*N* = 16, 3 men) were 21–47 years of age (mean 25 years). All subjects were right handed, had normal hearing, normal, or corrected-to-normal vision, and reported no history of psychiatric and neurological diseases or medications. Each subject signed an informed written consent before taking part in the experiment. The study protocol was approved by the Advisory Board on Research Ethics in Research with Human Participants, University of Helsinki.

### Stimuli and task

Auditory stimuli were pairs of iterated rippled noise bursts (16 iterations, delays 0.7–5 ms, corresponding pitch range 200–1400 Hz, equal mel steps). The sound pairs consisted of 90-ms parts (including 30-ms raised-cosine onset and offset ramps) with a 20-ms gap. The pitch difference between the first and second part of the pair was 9.5–95.5 mel depending on pitch sensitivity of the subject. Visual stimuli consisted of Gabor gratings (orientation 0–180°, step size 14.5°, duration 100 ms).

The auditory stimuli were delivered using Sensimetrics S14 insert earphones (Sensimetrics Corporation, USA). The noise of the scanner was attenuated by the insert earphones, circumaural ear protectors (Bilsom Mach 1) and viscous foam pads attached to the sides of the head coil. The visual stimuli were presented in the middle of a screen via a mirror fixed to the head coil.

The study consisted of six task conditions (auditory: precision, power, and no response; visual: precision, power, and no response) that were blocked and counterbalanced between the subjects. During all task conditions, subjects were presented with asynchronous streams of sound pairs (onset-to-onset 800–1000 ms, rectangular distribution, step 10 ms) and visual Gabor gratings (onset-to-onset interval 250–450 ms). The stimuli were presented in 12.5-s task blocks. Each block was followed by a 2-s response period, a 10-s period with no stimuli (“rest”), and a 4-s instruction period. During rest, subjects fixated on a cross presented in the middle of the screen (black on gray background). After 10 s, the fixation cross was replaced by a task symbol that remained on the screen until the end of the next task block.

During auditory task blocks, subjects were required to detect target sound pairs (50%) with increasing or decreasing pitch among non-target pairs with no pitch change. Depending on the task instruction, they responded immediately after detecting a target with a precision or a power grip or gave no motor responses. In half of the task blocks, there were more (70–75%) targets with increasing than decreasing pitch, whereas in the other half of the blocks there were more targets with decreasing pitch.

During visual task blocks, subjects detected changes in the orientation of the Gabor gratings. In half of the blocks, there were more (70–75%) targets with a clockwise (CW) change, whereas in the other half of the blocks there were more counterclockwise (CCW) orientation changes. The number of targets was identical in the visual and auditory tasks.

After each auditory block, an arrow (black on gray background) was presented in the middle of the screen for 2 s. The arrow pointed either up or down (equiprobably) indicating the question “there were more targets with increasing pitch change” or “there were more targets with decreasing pitch change,” respectively. Subjects were to answer this question by pressing the response button with their left index finger once (yes) or twice (no). After visual task blocks, subjects performed an identical task except that the arrow pointed either left or right (more targets with CCW or CW change, respectively).

Target responses were given using a modified joystick for precision grips (Current Designs, USA), grip force bar for power grips (Current Designs, USA), and a button on the joystick device for button presses. For precision grips, a custom-made piece of flexible plastic was attached to the body of the joystick device so that a precision grip response could be made by pinching the joystick shaft and the plastic plate together between the thumb and the middle/index finger of the right hand. Power grip responses were made by squeezing the grip force bar with the whole hand or with index finger and the thumb. A button press response was made with the left index finger. The joystick and the grip force bar were attached to a custom-made plastic frame that was placed on the subject's torso.

We reasoned that any sensorimotor modulations would be stronger if the motor task required selecting and executing one out of two response alternatives rather than continuously repeating the same response. Therefore, we trained our subjects to use slightly different grips (two or three-finger precision grip and two or five finger power grip) depending on the type of the target. During auditory precision-grip response tasks, one half of subjects responded with a two-finger pinch (fingertips of index finger and thumb) to targets with increasing pitch and with a three-finger pinch (fingertips of index finger, middle finger, and thumb) to targets with decreasing pitch, whereas the other half of subjects used the two-finger pinch for targets with decreasing pitch and three-finger pinch for targets with increasing pitch. Correspondingly, during auditory power grip tasks, half of the subjects used a two finger power grip (squeezing the power bar using the index finger and thumb) and a five-finger power grip to respond to targets with increasing and decreasing pitch, respectively, whereas for the other half of subjects this mapping was reversed. These responses were analogously used in the visual task to respond to CW and CWW Gabor orientation changes.

The experiment was conducted in one 35-min run. Each of the six conditions was repeated 12 times in random order. The experiment was controlled using Presentation software (Neurobehavioral Systems, USA).

### Pre-fMRI training

Before fMRI, each subject was carefully trained (two 1-h training sessions) to perform the auditory and visual tasks and, in particular, to correctly use the different grips for responding.

During the training, subjects were informed that the tasks were intentionally demanding and that maximal effort was required.

### Analysis of task performance

Performance was analyzed separately for auditory and visual tasks and for each grip type. Responses occurring 200–1300 ms from target onset were accepted as hits (irrespective of direction of change). Other responses (i.e., extra responses after a hit or outside the response window) were considered false alarms. Hit rate (HR) was defined as the number of hits divided by the number of targets. False alarm rate (FaR) was defined as the number of false alarms divided by the number of responses. HRs and FaRs were used to compute the index of stimulus detectability [*d*' = Z(HR) − Z(FaR)] and response bias [*c* = −0.5^*^[*Z*(*HR*)+*Z*(*FaR*)]]. Reaction times were calculated only for hits.

### fMRI data acquisition and analysis

fMRI data were acquired with a 3T MAGNETOM Skyra scanner (Siemens Healthcare, Erlagen, Germany) using a standard 20-channel head-neck coil. First, a high-resolution anatomical image (sagittal slices, slice thickness 1.0 mm, in-plane resolution 1.0 × 1.0 mm^2^) was acquired. Second, functional images (GE-EPI; TR 2220 ms, TE 30 ms, flip angle 78°, voxel matrix 96 × 96, FOV 18.9 cm, slice thickness 2.0 mm with no gap, in-plane resolution 2.0 × 2.0 mm^2^, 27 slices) were acquired. The middle EPI slices were aligned along the Sylvian fissures based on the anatomical image (see Figure 2 of Rinne et al., 2012). The imaged area covered the superior temporal lobe, insula, and most of the inferior parietal lobes in both hemispheres. Finally, a T2-weighted image using the same imaging slices but a denser in-plane resolution was acquired (TR 4500 ms, TE 100 ms, voxel matrix 256 × 256, FOV 18.9 cm, slice thickness 2.0 mm).

**Figure 1:**
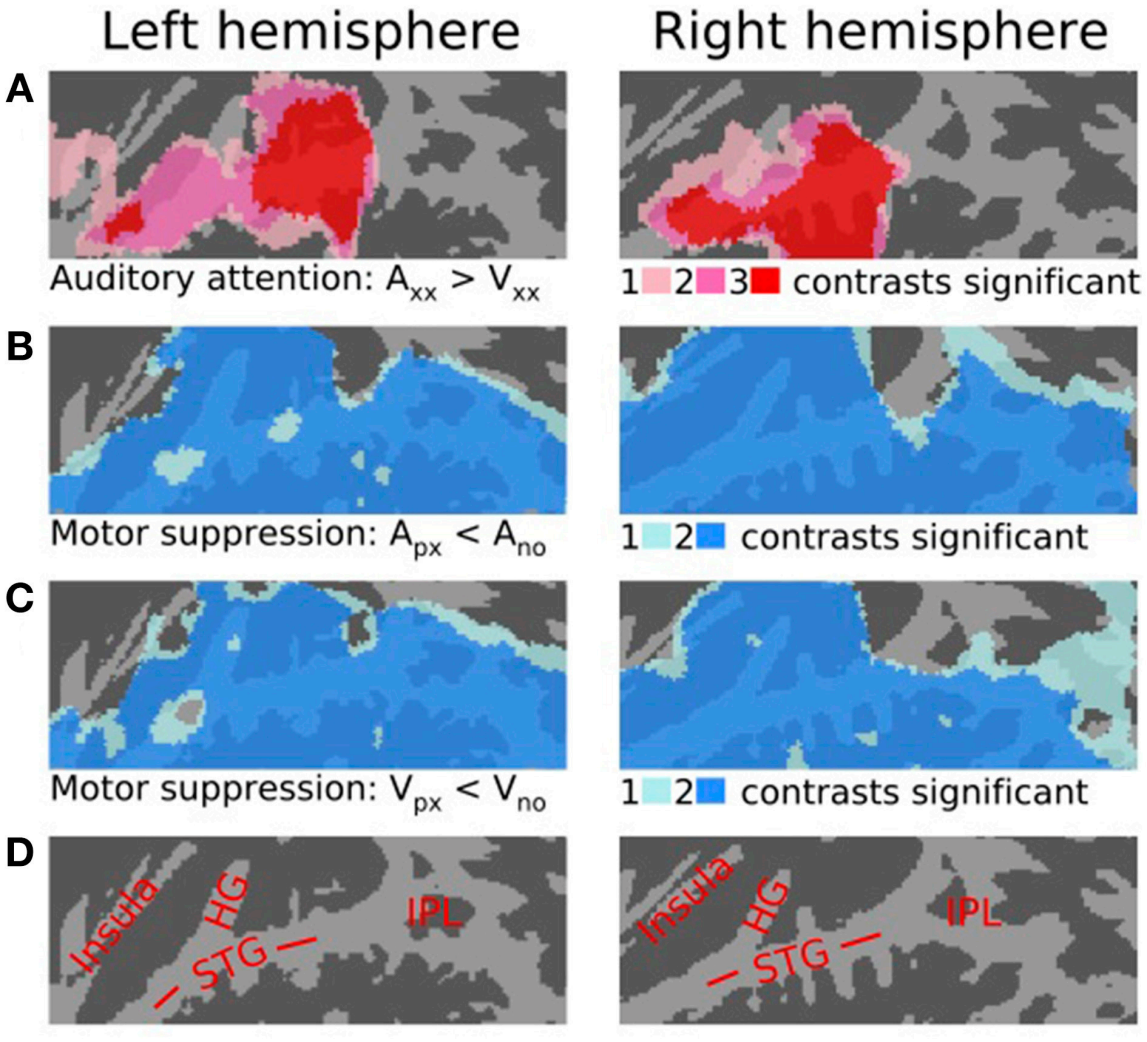
**The effects of auditory attention and motor responding on activations to sounds shown on a flattened mean 2D cortical surface (*N* = 16, corrected *P* < 0.05). (A)** The areas where activations were enhanced during the auditory task as compared with visual task with identical stimuli and motor responses. The results of three separate contrasts (A_pr_ > V_pr_, A_po_ > V_po_, A_no_ > V_no_) are plotted so that areas where any one of the contrasts was significant are shown in pink and areas where all three contrasts were significant are shown in red. **(B)** Motor suppression during the auditory tasks. The results of two contrasts (A_pr_ < A_no_, A_po_ < A_no_) are plotted so that areas where either of the contrasts was significant are shown in light blue and areas where both contrasts were significant are shown in darker blue. **(C)** Motor suppression during the visual task (V_pr_ < V_no_, V_po_ < V_no_). **(D)** Anatomical labels: STG, superior temporal gyrus; HG, Heschl's gyrus; PT, planum temporale; IPL, inferior parietal lobule.

Cortical surface reconstruction and coregistration were conducted using Freesurfer (version 5.3, www.freesurfer.net). Functional data were motion-corrected, resampled to the standard cortical surface, and surface-smoothed (10 mm FWHM). Global voxel-wise analysis was performed in surface-space, using FSL's (version 5.0.8; www.fmrib.ox.ac.uk/fsl) general linear model in which each task, the 2-s response period after each task block, and the 4-s instruction period before each task block were entered as separate explanatory variables. The hemodynamic response function was modeled with a gamma function (mean lag 6 s, SD 3 s) and its temporal derivate. Group analysis was performed using PALM (Permutation Analysis of Linear Models; version alpha26, Winkler et al., 2014). Significance was assessed by permutation inference (10,000 permutations). Correction for multiple comparisons (FWER) was performed using threshold-free cluster enhancement (tfce). For visualization, results were converted to 2D using Mollweide projection (Python libraries matplotlib and basemap, http://matplotlib.sourceforge.net, see Figure 1D).

### Regions of interest

**Figure 2:**
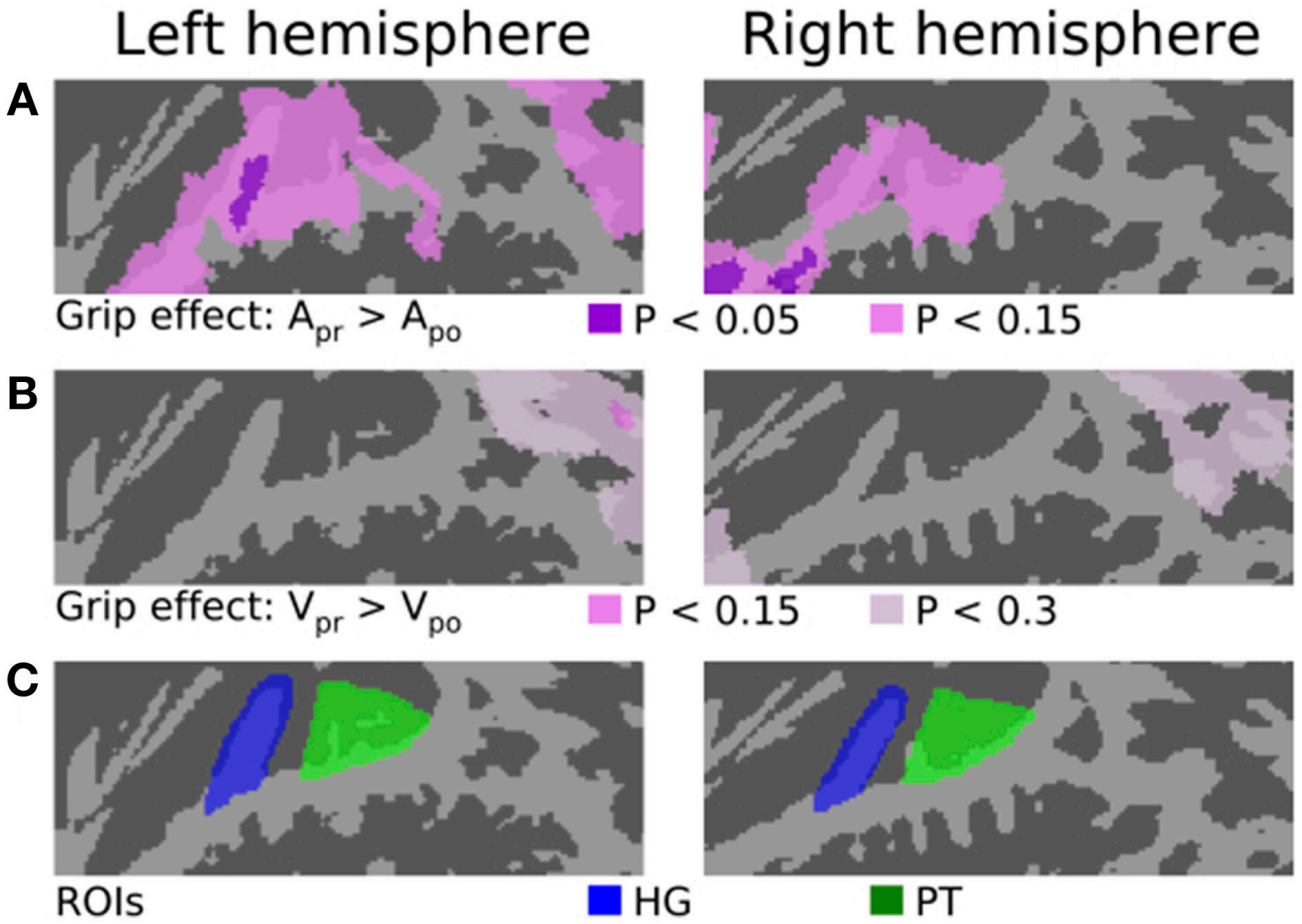
**The effect of grip and ROIs. (A)** The areas where activations were stronger during A_pr_ than A_po_ blocks. The results are plotted with two thresholds (corrected *P* < 0.05 and corrected *P* < 0.15). The opposite contrast (A_po_ > A_pr_) showed no significant effects. **(B)** The areas where activations were stronger during V_pr_ than V_po_ blocks (corrected *P* < 0.15 and corrected *P* < 0.3). (**C)** ROIs in HG and PT.

Two anatomical ROIs (Heschl's gyrus, HG and PT) were defined in the flattened 2D space for each hemisphere (see Figure 2C). One ROI was hand drawn in the 2D standard space to cover HG. The PT ROIs were identical to those used in our previous study (Hickok and Saberi, 2012; Rinne et al., 2014).

## Results

### Performance

After each task block, subjects (*N* = 16) reported the relative number of targets with increasing/decreasing pitch or with CW/CCW orientation change in the auditory (mean correct responses 76 ± 6% SEM) and visual (86 ± 5% SEM) tasks, respectively. Importantly, all subjects performed above chance level (50%) in both auditory and visual tasks. Performance was analyzed with a repeated measure ANOVA with two within-subject factors Modality (auditory, visual) and Response Type (precision grip, power grip, no response). Further, due to technical problems target responses were lost for four subjects and therefore, an additional between-subjects factor (target responses present/lost) was included. The ANOVA showed no significant main effects or interactions. This suggests that overall performance was equally good in auditory and visual tasks and that the type of motor response did not significantly affect target detection performance.

In addition, subjects responded to targets in 2/3 of the task blocks. Target detection performance (d', c, and reaction times, see Table 1) was examined using Two-Way repeated measures ANOVAs (*N* = 12) with factors Modality (auditory, visual) and Response (precision grip, power grip). The ANOVA for d' showed that performance was more accurate in the visual than auditory task [main effect of Modality, *F*_(1, 11)_ = 8.7, *p* < 0.01]. The ANOVA for response bias (C) showed a significant main effect of Modality [*F*_(1, 11)_ = 23.7, *p* < 0.001] suggesting that subjects used a more lenient decision threshold in the auditory than visual tasks. Also, the interaction between Modality and Response was significant [*F*_(1, 11)_ = 9.6, *p* < 0.01], due to subjects using a more lenient threshold during the auditory precision grip tasks than during the auditory power grip tasks. There was, however, no difference in C during the visual precision and power grip tasks. The ANOVA for RTs showed a significant main effect of Modality [*F*_(1, 11)_ = 68.6, *p* < 0.001]. RTs were shorter during the visual than auditory tasks consistent with the fact that information to detect a target was available later in the auditory (target could be detected at the beginning of the second part of the sound pair) than in the visual task (target could be detected at the beginning of the stimulus). In summary, the behavioral data acquired during fMRI indicate that subjects performed the tasks as instructed.

**Table 1:** **Mean (SEM) d', C, and RT in auditory task and visual tasks**.

**Task**		**Precision**	**Power**
Auditory	D'	2.0 (0.3)	2.2 (0.3)
	C	0.3 (0.1)	0.4 (0.1)
	RT	740 (30)	740 (30)
Visual	D'	2.8 (0.2)	3.0 (0.1)
	C	0.8 (0.1)	0.8 (0.1)
	RT	590 (30)	570 (30)

### fMRI

Consistent with many previous studies, activations to sounds were strongly modulated by attention (i.e., auditory task vs. visual task). Comparison between all auditory and all visual tasks (with identical stimuli and responses) showed stronger activations in wide STG regions during the auditory tasks. The results of three separate contrasts testing for auditory attention effects (auditory precision > visual precision, A_pr_ > V_pr_; auditory power > visual power, A_po_ > V_po_; auditory no response > visual no response, A_no_ > V_no_) are shown in Figure 1A. The results of these contrasts are plotted so that areas where any one of the contrasts was significant (corrected *P* < 0.05) are shown in pink, areas where any two contrasts were significant in darker pink, and areas where all three contrasts were significant in red. Note that auditory attention enhanced activations in similar areas in both hemispheres. Figure 1B shows the results of contrasts testing for motor suppression during the auditory task performed with or without target responses. The results of two contrasts (A_pr_ < A_no_, A_po_ < A_no_) are plotted so that areas where either one was significant are shown in light blue and areas where both contrasts were significant are shown in darker blue. Correspondingly, Figure 1C shows motor suppression during the visual task (V_pr_ < V_no_, V_po_ < V_no_). Note that decreased activations in wide temporal areas were similarly observed during both auditory and visual tasks.

Figure 2 shows the results of contrasts testing for the effects of motor grip type (precision vs. power). The first row shows areas where activations were stronger during A_pr_ than A_po_ blocks. The results are plotted with two thresholds. At corrected *P* < 0.05 (dark violet), a significant effect was observed in left lateral HG and also in right anterior STG and temporal pole. At a more lenient (non-significant) threshold (corrected *P* < 0.15; lighter violet), more widespread activation enhancements were observed in STG bilaterally and in left IPL. The opposite contrast (A_po_ > A_pr_) revealed no activation differences (corrected *P* < 0.3) associated with the power grip (not shown). The second row of Figure 2 shows the results of the corresponding visual contrast (V_pr_ > V_po_). During the visual task, no significant effects associated with grip type were detected. At a more lenient (non-significant) threshold (corrected *P* < 0.3), stronger activations during V_pr_ than V_po_ were observed bilaterally in IPL and in right temporal pole. Note that similar (non-significant) grip effects in IPL and temporal pole were observed during both auditory and visual tasks. These (non-significant) effects could be related to similar processing requirements during both tasks (e.g., as identical grips were used). However, in STG regions the grip effects, observed only during the auditory task, seem to arise from an interaction between motor- and auditory-task components.

### ROI analysis

**Figure 3:**
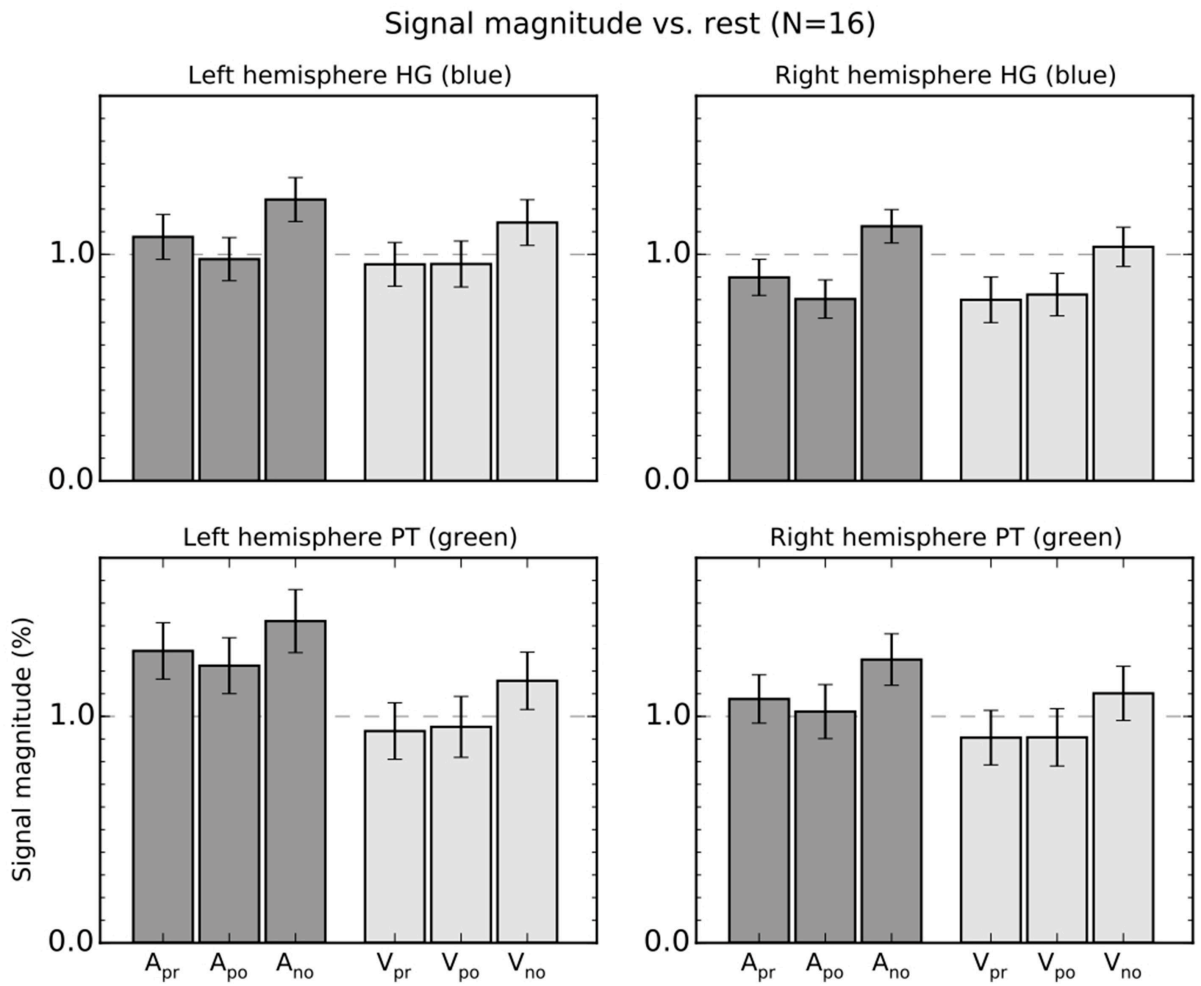
**Percentage signal magnitude in the left and right hemisphere planum temporale (PT) and Heschl's gyrus (HG) ROIs**. The bars show mean (±SEM) ROI signal relative to rest. The colors in panel titles refer to ROIs shown in Figure 2C.

We conducted a ROI analysis to investigate in more detail the activations in HG (primary AC) and PT (a non-primary auditory cortical area that has been previously linked with audiomotor integration; ROIs are shown in Figure 2C). Mean signal magnitudes (±SEM) in these ROIs during auditory (dark gray) and visual (lighter gray) tasks are shown in Figure 3.

Three-Way ANOVAs with the factors Modality (Auditory, Visual), Response (precision, power, no response), and ROI (HG, PT) were conducted separately for each hemisphere (unless otherwise stated, the reported *F*- and *P*-values are valid for identical tests conducted separately for left and right hemispheres). These ANOVAs showed significant main effects of Modality [*F*_(1, 15)_ > 12, *p* < 0.01] and Response [*F*_(1, 15)_ > 13, *p* < 0.001]. The interactions Modality × ROI [*F*_(1, 15)_ > 13, *p* < 0.01] and Modality × Response × ROI [*F*_(1, 15)_ > 4.5, *p* < 0.05] were also significant. Direct contrasts between auditory and visual tasks revealed that the difference between auditory and visual conditions was significant in HG [*F*_(1, 15)_ > 5.5, *p* < 0.05] and PT [*F*_(1, 15)_ > 17, *p* < 0.001]. However, the two-way interaction emerged because the difference between the auditory and visual tasks was larger in PT than HG [*F*_(1, 15)_ > 12, *p* < 0.01]. The three-way interaction was observed because, first, signal magnitudes in HG were higher during A_pr_ than A_po_ blocks [*F*_(1, 15)_ > 6.0, *p* < 0.05]. Second, no significant differences were observed during the visual task [V_pr_ vs. V_po_, *F*_(1, 15)_ < 0.3]. Third, in PT the difference between A_pr_ and A_po_ task blocks was smaller than in HG and was significant only in the left hemisphere [left hemisphere, *F*_(1, 15)_ = 4.9, *p* < 0.05; right hemisphere, *F*_(1, 15)_ = 2.3].

Taken together, the two- and three-way interactions reveal that the effect of auditory attention on signal magnitudes was stronger in PT than in HG, whereas the effect of grip on signal magnitudes during the auditory tasks was stronger in HG than in PT.

## Discussion

The present study investigated how motor responding modulates activations to sounds in AC. We presented identical stimuli during demanding auditory and visual discrimination tasks in which subjects reported the relative number of two different targets at the end of each task block. In addition, depending on the task instruction, they also responded to each target using a precision grip, power grip, or gave no motor responses. First, in line with a large number of previous studies (Hall et al., 2000; Petkov et al., 2004; Rinne et al., 2009, 2012; Rinne, 2010), we found that activations in regions extending from anterior to posterior STG were modulated by attention (stronger activations during the auditory than visual tasks). Second, activations in wide STG regions decreased when subjects responded to targets using precision and power grips. This decrease of activations was similarly observed during both auditory and visual tasks. Third, we also found that AC activations were modulated by grip type during the auditory but not during the visual tasks.

### Suppression of AC activations during manual motor responding

Previous studies have shown that activations in AC to subjects' own voice are suppressed during vocalization (Curio et al., 2000; Houde et al., 2002; Eliades and Wang, 2003; Flinker et al., 2010; Greenlee et al., 2011; Agnew et al., 2013). This effect is believed to be caused by active suppression of the predicted sensory consequences of one's own vocalization (corollary discharge).

Similar suppression effects have also been observed in response to self-triggered vs. externally triggered non-vocal sounds (Martikainen et al., 2005; Baess et al., 2008, 2009, 2011; Aliu et al., 2009; SanMiguel et al., 2013; Timm et al., 2013). In the present study, AC activations were suppressed when subjects made manual responses to targets in a computer-controlled stream of noise bursts. In contrast to previous studies showing suppression to subjects' own vocalization or self-initiated sounds, the motor responses in the present study did not cause or trigger the presentation of the sounds. In particular, any motor-auditory links were fully absent during the visual task in which subjects directed their attention to visual stimuli, responded to visual targets, and ignored the task-irrelevant and asynchronous sounds. As the suppression effect was observed during both auditory and visual tasks, it is evident that the suppression is directly related to motor responding and not to an association between a motor act and its predicted sensory consequences.

A recent study reported that the spontaneuous and tone-evoked activity of excitatory neurons in rat AC is suppressed before and during a wide range of natural movements (e.g., locomotion and head movements; Schneider et al., 2014). Using electrophysiology and optogenic methods it was shown that these effects arise directly from signals from motor cortex to AC during movement and that the suppression is likely to be due to direct motor-related signals rather than sensory reafference. The present study extends these results by showing that a similar general suppression is present in human AC during manual movements.

It could perhaps be argued that the present AC suppression effects were actually due to a difference in attentional demands between the response and no-response conditions. This is an important point to consider, as any small differences in the allocation of attention could easily be associated with strong activation modulations in AC (Alho et al., 2014). According to this argument, AC activations were weaker during motor responding as, in addition to auditory stimuli, attention had to be allocated to the motor task. However, differences in auditory attention do not easily explain the result that a similar suppression effect in AC was observed also during the visual task, given that the visual task did not require auditory attention, motor responses were related to visual and not to auditory targets, and subjects were engaged in the same demanding and attention-engaging task during both response and no-response blocks. In addition, our previous study showed that increasing the difficulty of a visual task did not significantly modulate AC activations to sounds (Rinne, 2010). Thus, it is very unlikely that the strong AC suppression effects observed during the visual task could be explained by fluctuating auditory attention. Further, the results of recent studies investigating auditory event-related potentials to self-initiated sounds suggest that the suppression of AC activations (N1 component) is independent of attention (Saupe et al., 2013; Timm et al., 2013). Taken together, it is very unlikely that attention-related modulation of activations explain the present AC suppression effects.

### The effect of grip type

In addition to the attention and motor suppression related effects, we also found that activations in STG regions to sounds were stronger when target responses were made with a precision rather than a power grip. This difference was observed during the auditory but not during the visual task. In IPL, enhanced activations associated with the precision grip were observed during both auditory and visual tasks (non-significant effect). It could be argued that the precision and power grips have different requirements for accuracy. During a precision grip, force is applied between the fingertips of isolated digits to a small target, whereas a power grip demands the whole-hand for higher stability and power around a larger object. Precision grips may therefore pose higher demands for integration between motor commands and somatosensory feedback to enable spatially accurate performance (Ehrsson et al., 2000). IPL has been implicated in both sensorimotor integration and spatial processing (Grefkes and Fink, 2005). Thus, the present enhanced IPL activations during precision blocks could be because the precision grip required more refined neural control than the power grip.

This account, however, does not explain the result that stronger STG activations were observed during the precision responses only during the auditory task. One possibility is that the activation differences in STG between the A_pr_ and A_po_ blocks were caused by direct or indirect modulation of auditory attention. The A_*pr*_ blocks could have been more demanding resulting in increased auditory attention-related activations in STG, or the execution of the power grips could have required more motor attention resulting in decreased auditory attention. While this idea cannot be fully dismissed, two pieces of evidence speak against it. First, in both A_pr_ and A_po_ blocks, subjects performed the same demanding task and reported the relative number of targets with increasing/decreasing pitch at the end of the block. There were no significant performance differences in this task between A_pr_ and A_po_ blocks, suggesting that the grip used for target responses did not modulate task difficulty. The lack of significant differences in target performance between A_pr_ and A_po_ blocks also supports this conclusion. Second, while the grip effect was observed in similar regions as the auditory attention effect, the results of the ROI analysis indicate that auditory attention and grip modulate activations differently in HG and PT. Thus, the activation differences in STG between A_pr_ and A_po_ blocks appear to be distinct from attention-related effects.

It is also possible that the activation differences between the A_pr_ and A_po_ blocks were due to response-specific suppression. According to this account, activations in AC are modulated by both unspecific (observed during both tasks, see Section Suppression of AC Activations during Manual Motor Responding) and specific motor suppression (observed only during auditory tasks) due to general gating of AC activations and predicting the specific sensory consequences of movements during motor responding, respectively (Horváth et al., 2012; Horváth, 2014; Schröger et al., 2015). In the present study no distinct sounds were associated with the precision and power grips. However, it is possible that the activation differences between A_pr_ and A_po_ blocks are due to specific motor suppression based on pre-learnt motor-auditory associations.

An alternative perspective is provided by the idea that the motor system involved in grasping may have been in a central role during the evolution of language (Hewes, 1973; Gentilucci and Corballis, 2006). Thus, motor programs for manual grips may be strongly linked with auditory operations. It could be speculated that such links between manual motor programs and AC could interact with the processing of pitch information during a demanding auditory task. There might also be selective modulatory connections between motor and auditory cortices that are used to monitor and fine-tune auditory processing in a motor context (Warren et al., 2005; Hickok and Poeppel, 2007; Hickok, 2010; Hickok et al., 2011). Such functional links between motor and auditory processing could underlie the present grip effects.

### Conclusion

The connections between motor and auditory cortices play a key role in current auditory models (Warren et al., 2005; Hickok and Poeppel, 2007; Zatorre et al., 2007; Rauschecker and Scott, 2009; Rauschecker, 2010). It is assumed that motor cortex provides AC with predictive information about upcoming sounds during speech production or other sound producing activity (Buchsbaum et al., 2001; Rauschecker and Scott, 2009; Rauschecker, 2010; Hickok et al., 2011; Schröger et al., 2015). In the present study, motor responding was not associated with sound production, and any sounds resulting from motor responding were most likely inaudible and effectively masked by the loud and continuous fMRI scanner noise. Nevertheless, we found clear motor suppression and grip effects in AC. Together the present findings are consistent with the view that motor signals may directly modulate operations in human AC.

The functional role of motor-auditory links are still not fully understood. The present study demonstrates three important factors that should be considered in subsequent studies. First, as AC activations are strongly modulated by attention, even slight differences in attentional demands could easily result in attention-related effects. Attention-related modulations are particulary strong in areas that are also implicated in audiomotor integration (e.g., PT). Therefore, attention- and task-related factors should be carefully controlled in studies on audiomotor integration. Second, AC activations are also strongly affected by motor suppression. If not specifically taken into account, the motor suppression effect could easily confound comparisons across different conditions. Third, in addition to previous studies showing audiomotor effects in AC during sound production, our results suggest that motor input may modulate AC activations in a general (no auditory-motor link) or a task-specific (behaviorally relevant auditory-motor link) manner.

### Conflict of interest statement

The authors declare that the research was conducted in the absence of any commercial or financial relationships that could be construed as a potential conflict of interest.
